# Giant Congenital melanocytic nevus with expanded proliferating nodules in a Syrian neonate

**DOI:** 10.1093/omcr/omab040

**Published:** 2021-06-18

**Authors:** Leen Jamel Doya, Hanin Ahmed Mansour, Naya Talal Hassan, Nadim Jouni, Oday Jouni

**Affiliations:** 1 Department of Pediatrics, Tishreen University Hospital, Faculty of Medicine, Latakia, Syria; 2 Department of Pediatrics, Tishreen University Hospital, Latakia, Syria; 3 Department of Dermatology, Tishreen University Hospital, Latakia, Syria; 4 Department of Anesthesia, Tishreen University Hospital, Latakia, Syria; 5 Professor of Neonatology, Department of Pediatrics, Tishreen University Hospital, Lattakia, Syria

**Keywords:** Giant congenital melanocytic nevus, neonate, hyperpigmented patches

**Figure 1:**
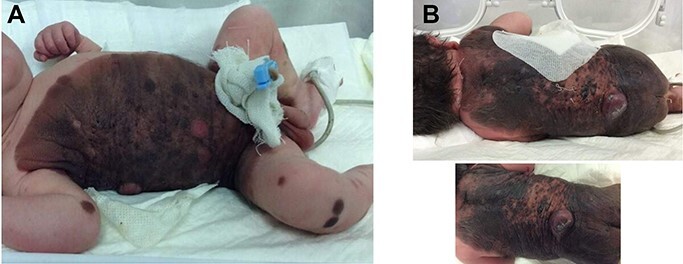
(**a**) Extensive hyperpigmented contained irregularly shaped over chest, abdominal wall, the suprapubic, inguinal and upper left thigh region with multiple pigmented satellite lesions over the extremities. (**b**) Hyperpigmented lesion over the posterior neck, back and buttocks.

A 3160 g full-term male baby was born via the cesarean section without any complications. At birth, extensive hyperpigmented contained irregularly shaped with large nodules in the supra-gluteal cleft and lateral part of the skin. The lesions were variegated colors, including grayish, blackish and light-to-dark brownish plaques covered 50% of the skin surface area distributed throughout his body with abundant hair (Fig. 1a and b). Multiple pigmented satellite lesions of size (4–5) cm were also present over the extremities. There were no other associated congenital anomalies. MRI of the spine and brain showed no deep CNS extension. The histology report from satellite and giant lesions confirmed giant congenital melanocytic nevus (GCMNs) with expanded proliferating nodules. Melanocytic cells were positive for S100, NSE, melancoktail, Ki67 in the superficial and deep component with negative for EMA, Desmin, and SMA. The parents refused any treatment at this age. Therefore, yearly continuous monitoring was recommended with special attention to changes in shape, texture, irregular borders, color variegation, symptoms like itching or bleeding. During a follow-up period of 2 years, this child remainswell.

To our knowledge, This is the first case of GCMNs to be reported in Syria. GCMNs are benign pigmented skin disorders more than 20.0 cm that is typically present at, or shortly after birth [1]. It has clinical significance as it is associated with the development of melanoma and neurological disturbances [2]. The activation of mutations in Five genes may constitute a risk factor for developed cutaneous melanoma (N-RAS, p53, p16, CDK4, MC1R) in GCMNs patients [3]. There are several procedures includes carbon dioxide laser, YAG, and Q-switched ruby laser for resurfacing and selectively treating the deep pigmentations. Besides, surgical treatment at the age of 6 months of GCMN by serial excision and reconstruction with skin grafting, tissue expansion, local rotation flaps, and free tissue transfer [4].

## References

[1] Viana ACL, Gontijo B, Bittencourt FV. Giant congenital melanocytic nevus. An Bras Dermatol 2013;88:863–78. doi: 10.1590/abd1806-4841.20132233. 24474093 PMC3900335

[2] Charbel C, Fontaine RH, Malouf GG, Picard A, Kadlub N, el-Murr N et al. NRAS mutation is the sole recurrent somatic mutation in large congenital melanocytic nevi. Journal of Investigative Dermatology 2014;134:1067–74. doi: 10.1038/jid.2013.429. 24129063

[3] Park SH, Koo SH, Choi EO. Combined laser therapy for difficult dermal pigmentation: resurfacing and selective photothermolysis. Ann Plast Surg 2001;47:31–6. doi: 10.1097/00000637-200107000-00006. 11756800

[4] Strauss RM, Newton Bishop JA. Spontaneous involution of congenital melanocytic nevi of the scalp. J Am Acad Dermatol 2008;58:508–11. doi: 10.1016/j.jaad.2006.05.076. 18280354

